# Supplementary material for: T cell receptor signaling strength establishes the chemotactic properties of effector CD8+ T cells that control tissue-residency
Source: Nat Commun. 2023 Jul 4;14:3928. doi: 10.1038/s41467-023-39592-1 (PMC10319879; doi:10.1038/s41467-023-39592-1)
Supplement: Supplementary file 1 — Supplementary Information [file 41467_2023_39592_MOESM1_ESM.pdf]

Supplementary Information

T cell receptor signaling strength establishes the chemotactic properties of effector $CD8^+$ T cells that control tissue-residency

Mahmoud Abdelbary, Samuel J. Hobbs, James S. Gibbs,
Jonathan W. Yewdell, and Jeffrey C. Nolz

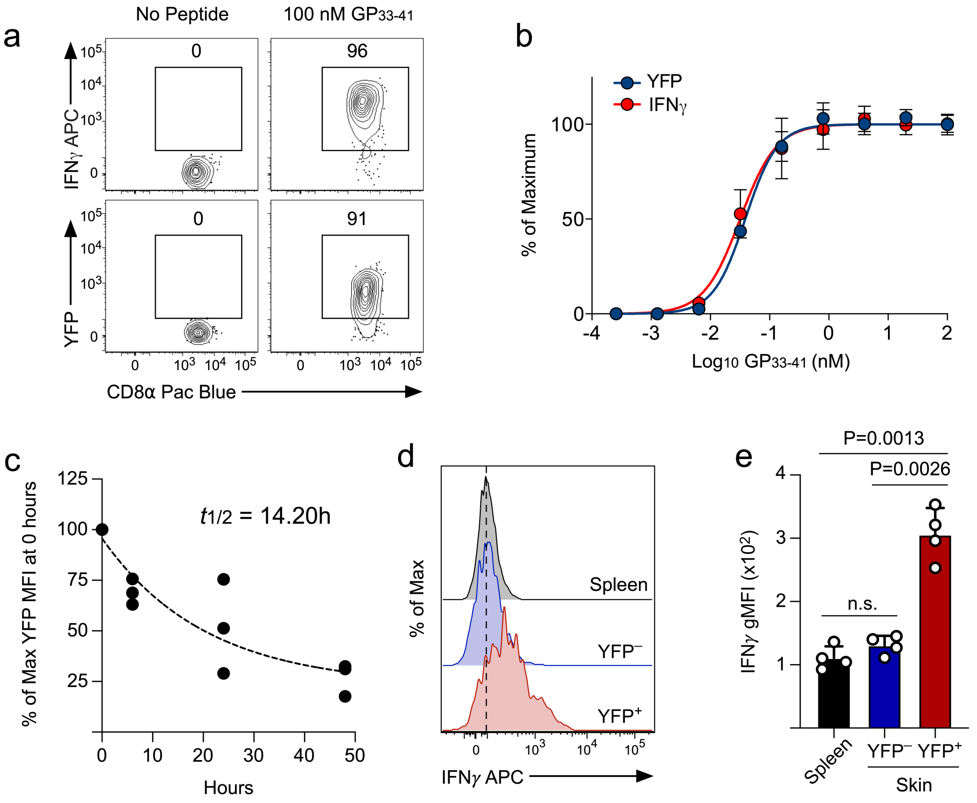

Supplementary Figure 1: YFP expression faithfully reports IFNγ expression by effector CD8$^+$ T cells. (a,b) Naive IFNγ-YFP P14 CD8$^+$ T cells were transferred into B6 mice and were infected with VacV-GP33 on the left ear skin. **(a)** Representative flow cytometry plots depicting YFP and IFNγ protein expression by IFNγ-YFP P14 CD8$^+$ T cells following stimulation with GP$_{33-41}$. **(b)** Quantification of (a) over a range of peptide concentrations; *n*=3 **(c)** Calculation of the approximate half-life of YFP following stimulation overnight with 100 nM GP$_{33-41}$ using a one phase decay curve; *n*=3 per time point. **(d,e)** Naive IFNγ-YFP P14 CD8$^+$ T cells were transferred into B6 mice and were infected with VacV-GP33 on the left ear skin. On day 5 post infection, mice were intravenously injected with Brefeldin A (250 µg/mouse) and were euthanized 18 hours later. **(d)** Representative histograms depicting IFNγ protein expression in YFP$^+$, YFP$^-$ and spleen effector P14 CD8$^+$ T cells. **(e)** Quantification of (d); *n*=4. Statistical significance (e) was calculated using one-way ANOVA followed by Tukey's multiple comparisons test. Source data are provided as a Source Data file.

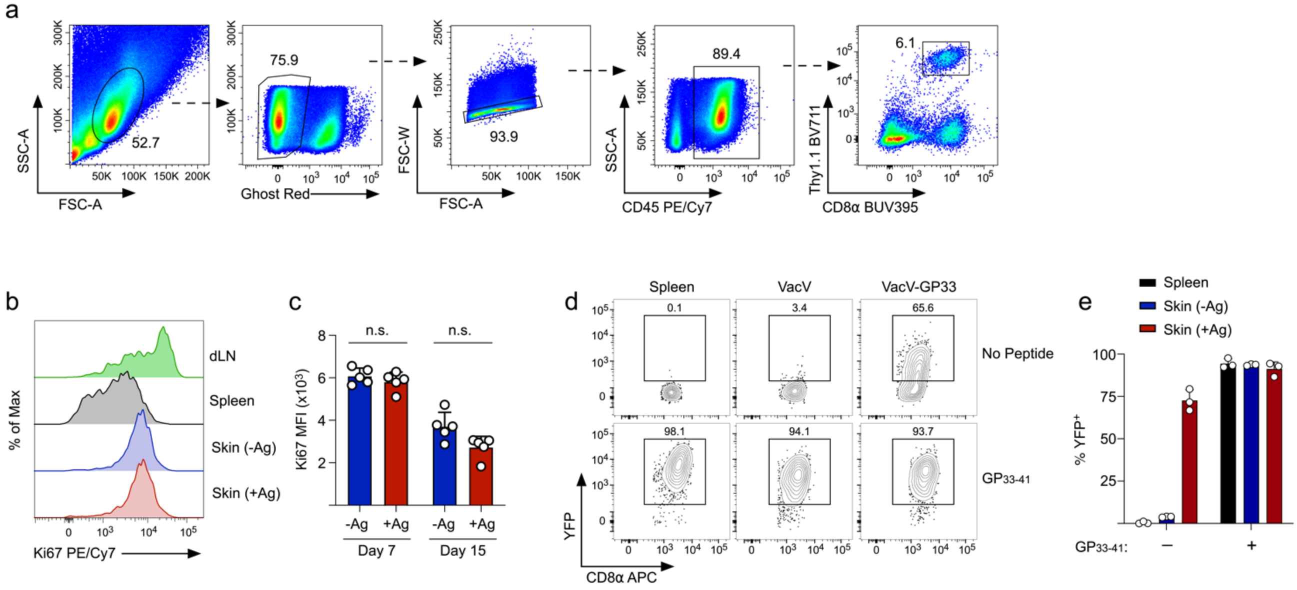

Supplementary Figure 2: Antigen recognition regulates IFNγ expression, but not proliferation of effector $CD8^+$ T cells in the skin. (a) Gating strategy to identify Thy1.1 P14 $CD8^+$ T cells in the skin. **(b)** Representative histograms depicting Ki67 expression in IFNγ-YFP P14 $CD8^+$ T cells in left (+Ag) and right (-Ag) skin, spleen and draining lymph node (dLN). **(c)** Quantification of (b); *n*=5. **(d)** Representative flow plots of YFP expression by IFNγ-YFP P14 $CD8^+$ T isolated from the spleen and skin (+/- Ag) following overnight incubation with 100 nM GP_{33-41}. **(e)** Quantification of (d); *n*=3. Data shown are mean ± SD and are representative of 2 or more independent experiments. Statistical significance was calculated using a two-sided paired t test (c) or one-way ANOVA followed by Tukey's multiple comparisons test (e). Source data are provided as a Source Data file.

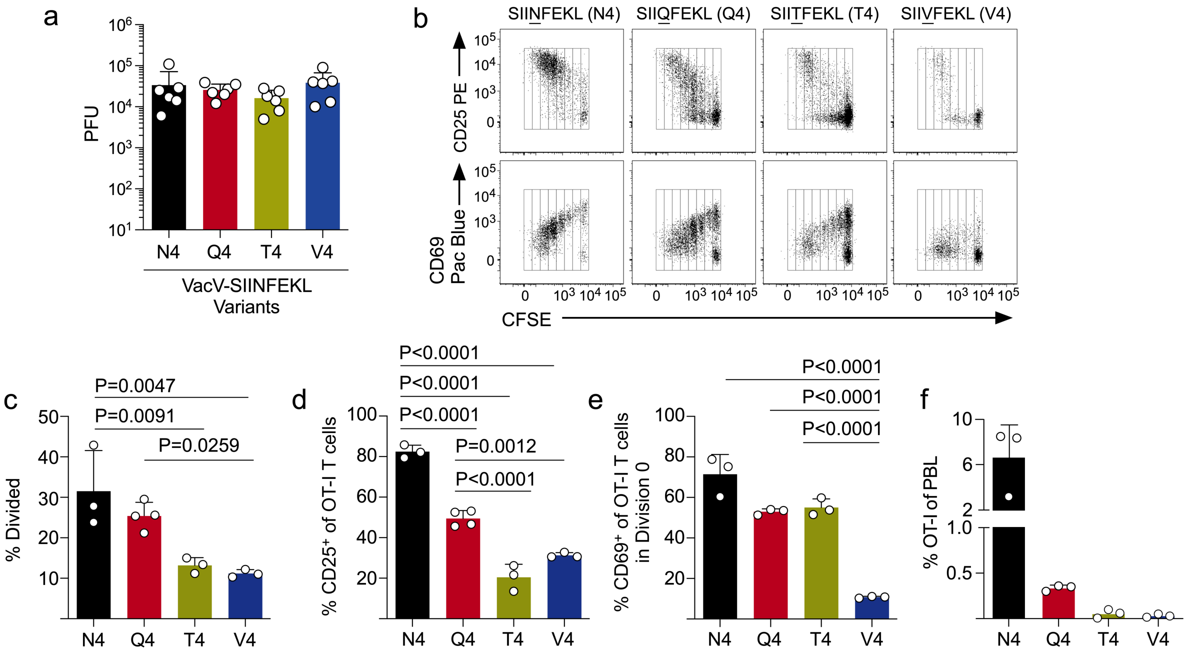

Supplementary Figure 3: Characterization of the OT-I CD8^{+} T cell responses by VacV expressing lower affinity SIINFEKL variants. Naïve OT-I CD8^{+} T were labelled with CFSE and transferred into B6 mice followed by infection with VacV expressing SIINFEKL variants on the left ear skin. The skin and draining lymph node were harvested on day 2 post infection. **(a)** Viral burden of the skin was quantified on day 2 post infection; *n*=6. **(b)** Representative flow plots depicting CFSE dilution, CD69 expression, and CD25 expression by OT-I CD8^{+} T cells in the draining lymph nodes. **(c-e)** Quantification of data shown in (b); *n*=3 or 4 per group as indicated. **(f)** Frequencies of OT-I CD8^{+} T cells in the peripheral blood on day 7 post-infection; *n*=3 per group. Data shown are mean ± SD and are representative of 2 independent experiments. Statistical significance (c-e) was calculated using a one-way ANOVA followed by Tukey's multiple comparisons test. Source data are provided as a Source Data file.

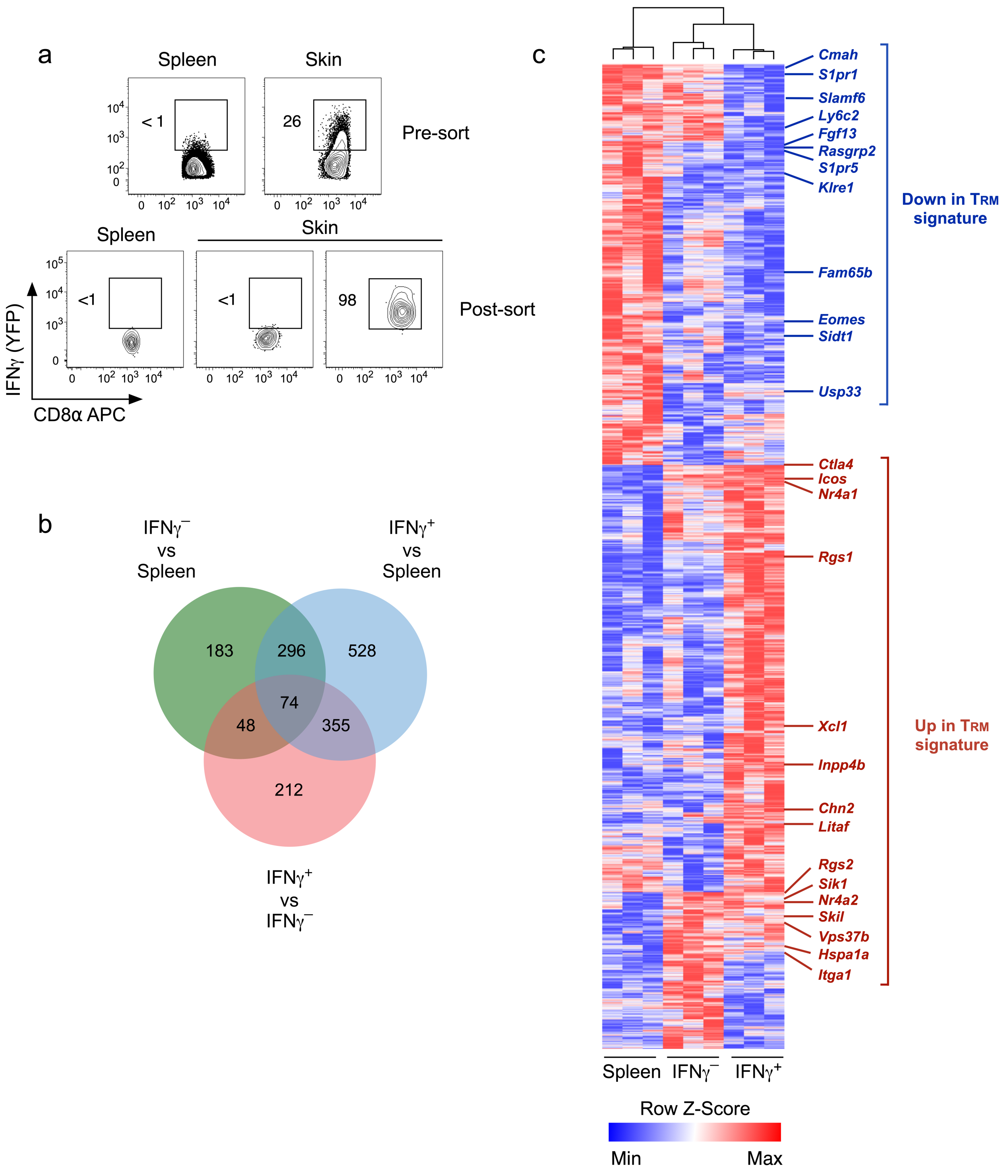

Supplementary Figure 4: The transcriptional profiles of IFNγ^{+} and IFNγ^{-} effector CD8^{+} T cells from the skin. Naïve IFNγ-YFP P14 CD8^{+} T cells were transferred into B6 mice and were infected on the left and right ear skin with VacV-GP33. On day 7 post infection, IFNγ^{+} and IFNγ^{-} effector P14 CD8^{+} T cells were sorted from the skin and spleen based on YFP expression. **(a)** Post-sort purity of samples used for gene expression analysis. **(b)** Venn diagram depicting the number of differentially expressed genes that are shared between each pairwise comparison. **(c)** Heatmap displaying relative expression levels of differentially expressed genes between the three sorted populations. Genes that are up- or down-regulated in the T$_{RM}$ core signature (Ref. 14) are labeled.

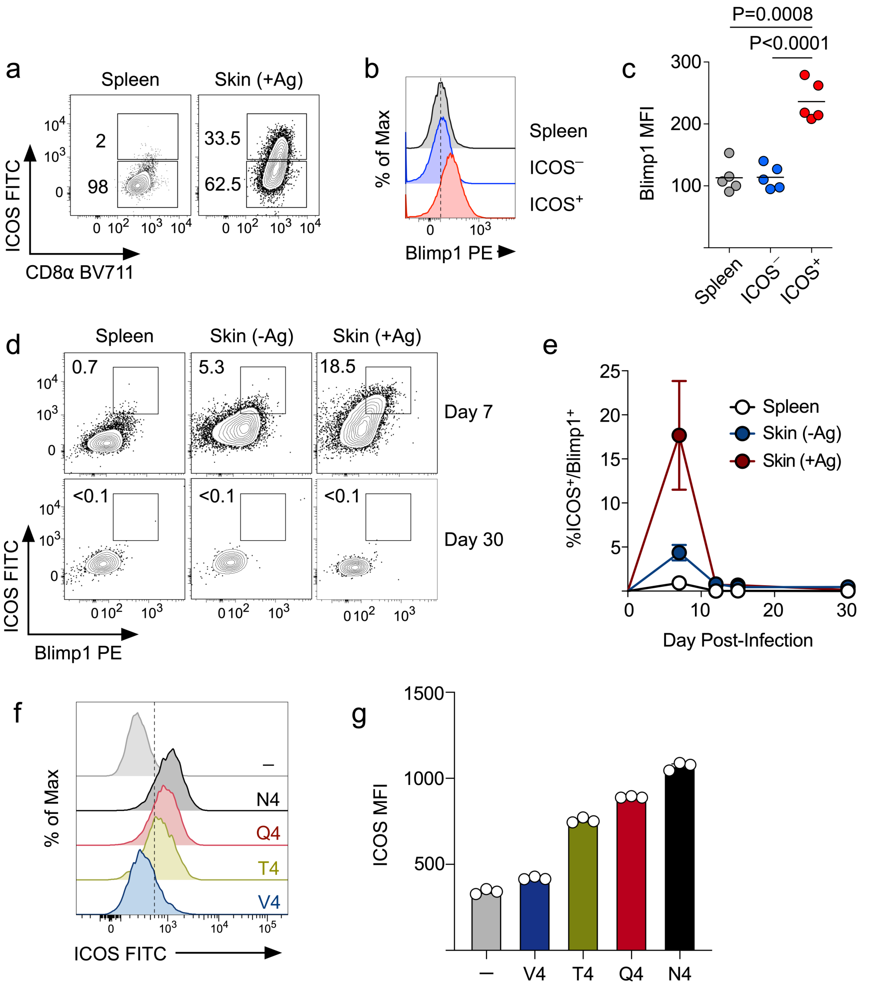

Supplementary Figure 5: Effector CD8$^+$ T cells express ICOS and Blimp1 in an antigen-dependent manner during viral skin infection. (a-d) Naïve P14 CD8$^+$ T cells were transferred into B6 mice and were infected with VacV-GP33 on the left ear skin. **(a)** Representative flow plots depicting ICOS expression by effector P14 CD8$^+$ T cells within the spleen and skin on day 7 post-infection. **(b)** Representative histograms depicting the expression of Blimp1 by the indicated subsets of effector P14 CD8$^+$ T cells in (a). **(c)** Quantification of (b); *n*=5. **(d)** Representative flow plots depicting co-expression of Blimp-1 and ICOS by effector P14 CD8$^+$ T cells in the spleen and skin at the indicated time points post infection. **(e)** Quantification of (d); *n*=5, 4, 3 and 5 for days 7, 12, 15 and 30, respectively. **(f,g)** Naïve OT-I CD8$^+$ T cells were transferred into B6 mice and were infected with VacV-SIINFEKL on the left ear skin. On day 10 post-infection, total splenocytes were stimulated ex vivo with 10 nM SIINFEKL variants. **(f)** Representative histograms depicting the expression of ICOS by OT-I CD8$^+$ T cells following stimulation with the indicated peptides. **(g)** Quantification of (f); *n*=3. Data shown are mean ± SD and are representative of 2 independent experiments. Statistical significance (c) was calculated using a one-way ANOVA followed by Tukey's multiple comparisons test. Source data are provided as a Source Data file.

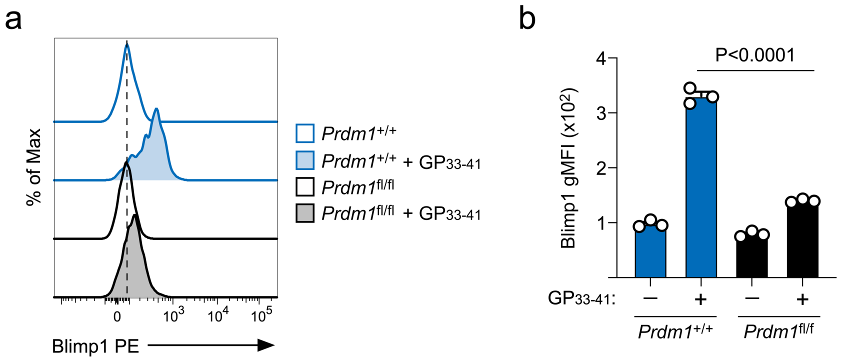

Supplementary Figure 6: Efficiency of Blimp1 deletion with tamoxifen. An ~1:1 mixture of naïve Thy1.1/Thy1.1 *Prdm1*$^{+/+}$ ROSA26-Cre-ERT2^{+} and Thy1.1/1.2 *Prdm1*$^{fl/fl}$ ROSA26-Cre-ERT2^{+} P14 CD8^{+} T cells were transferred into B6 mice and were co-infected with VacV-GP33 and VacV on the left and right ear skin, respectively. Tamoxifen treatment was initiated one day prior to the transfer of T cells and continued for 5 consecutive days as shown in Figure 6a. **(a)** Representative histograms depicting Blimp1 expression by *Prdm1*$^{+/+}$ ROSA26-Cre-ER^{T2+} and *Prdm1*$^{fl/fl}$ ROSA26-Cre-ER^{T2+} P14 CD8^{+} T cells following overnight stimulation with 1000 nM GP$_{33-14}$ peptide. **(b)** Quantification of (a); *n*=3. Data shown are mean ± SD and are representative of 2 independent experiments. Statistical significance (b) was calculated using a one-way ANOVA followed by Tukey's multiple comparisons test. Source data are provided as a Source Data file.

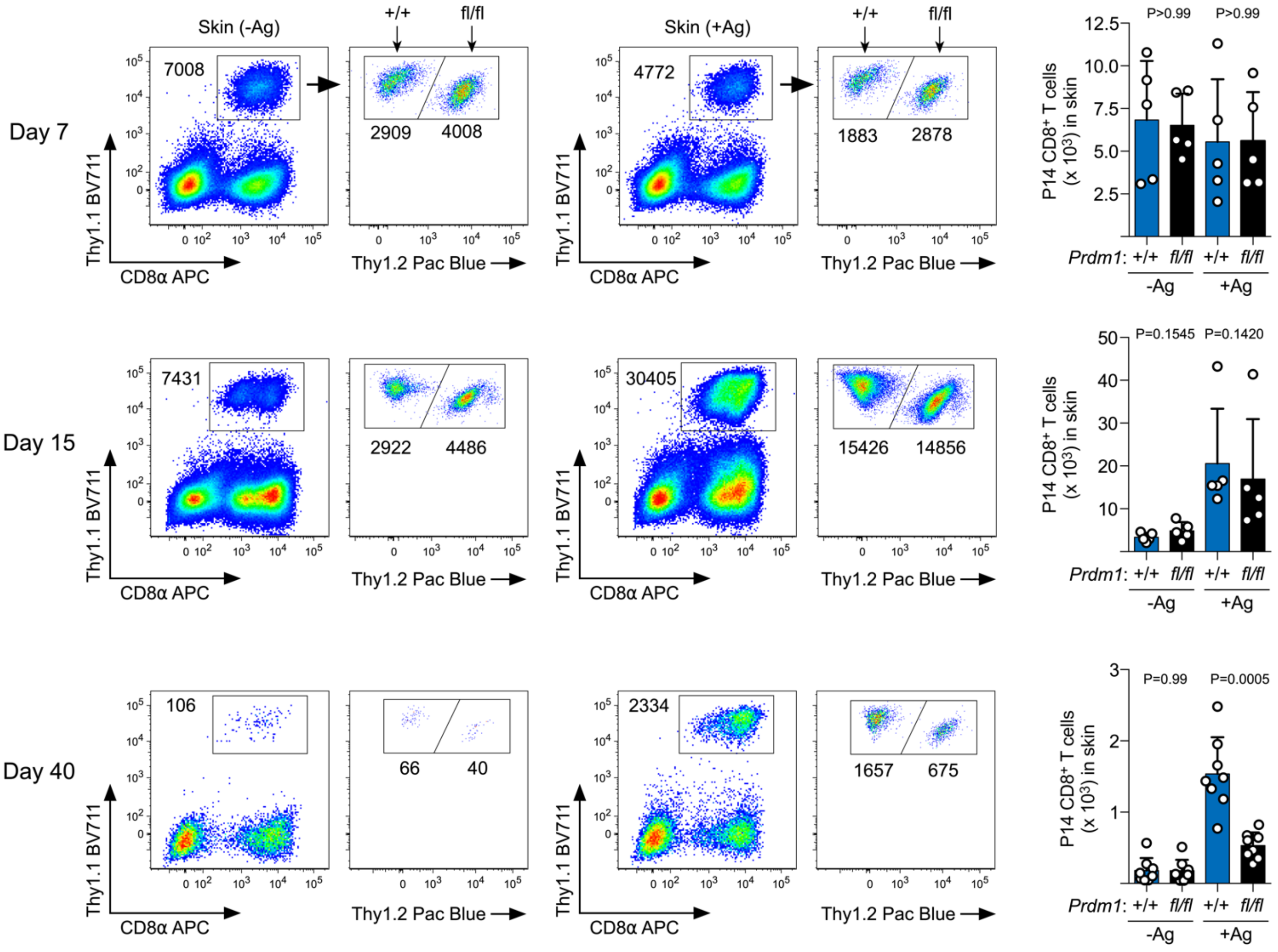

Supplementary Figure 7: Antigen recognition and Blimp1 are required for T_{RM} CD8$^+$ T cell formation in the skin during viral infection. An ~1:1 mixture of naïve Thy1.1/Thy1.1 *Prdm1*$^{+/+}$ ROSA26-Cre-ERT2$^+$ and Thy1.1/1.2 *Prdm1*$^{fl/fl}$ ROSA26-Cre-ERT2$^+$ P14 CD8$^+$ T cells were transferred into B6 mice and were co-infected with VacV-GP33 and VacV on the left and right ear skin, respectively. Tamoxifen treatment was initiated one day prior to the transfer of T cells and continued for 5 consecutive days as shown in Figure 6a. Representative flow cytometry plots and quantifications were performed on day 7, 15, and 40 post-infection; *n*=5 (days 7 and 15) or *n*=8 (day 40) per group. Data shown are mean ± SD and are representative of 2 independent experiments. Statistical significance was calculated using a one-way ANOVA followed by Tukey's multiple comparisons test. Source data are provided as a Source Data file.

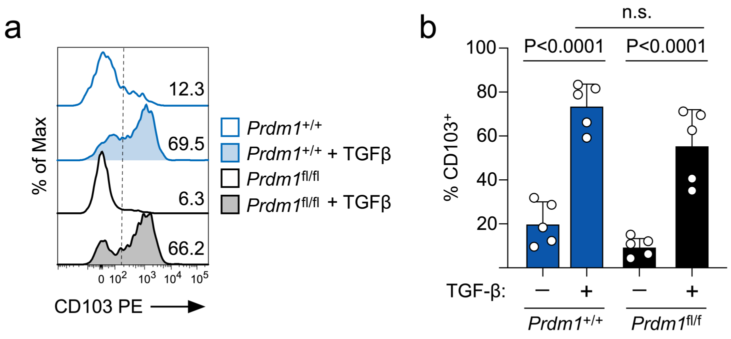

Supplementary Figure 8: Blimp1 is not required for TGF-β-stimulated expression of CD103. (a) Representative histograms depicting CD103 expression by *Prdm1*$^{+/+}$ ROSA26-Cre-ERT2^{+} and *Prdm1*$^{fl/fl}$ ROSA-26-Cre-ERT2^{+} P14 CD8^{+} T cells on day 10 post-infection as described in Figure 6a following incubation with TGF-β (10 ng/ml) for 48 hours. **(b)** Quantification of (a); *n*=5 per group. Data shown are mean ± SD and representative of 2 independent experiments. Statistical significance (b) was calculated using a one-way ANOVA with repeated measures followed by Tukey's multiple comparisons test. Source data are provided as a Source Data file.
