# Supplementary material for: T cell receptor signaling strength establishes the chemotactic properties of effector CD8+ T cells that control tissue-residency
Source: Nat Commun. 2023 Jul 4;14:3928. doi: 10.1038/s41467-023-39592-1 (PMC10319879; doi:10.1038/s41467-023-39592-1)
Supplement: Supplementary file 3 — Reporting Summary [file 41467_2023_39592_MOESM3_ESM.pdf]

nature portfolio

Corresponding author(s): Jeffrey C. Nolz

Last updated by author(s): 6-7-2023

Reporting Summary

Nature Portfolio wishes to improve the reproducibility of the work that we publish. This form provides structure for consistency and transparency in reporting. For further information on Nature Portfolio policies, see our Editorial Policies and the Editorial Policy Checklist.

Statistics

For all statistical analyses, confirm that the following items are present in the figure legend, table legend, main text, or Methods section.

n/a	Confirmed	
☐	☒	The exact sample size (*n*) for each experimental group/condition, given as a discrete number and unit of measurement
☐	☒	A statement on whether measurements were taken from distinct samples or whether the same sample was measured repeatedly
☐	☒	The statistical test(s) used AND whether they are one- or two-sided<br>*Only common tests should be described solely by name; describe more complex techniques in the Methods section.*
☐	☒	A description of all covariates tested
☐	☒	A description of any assumptions or corrections, such as tests of normality and adjustment for multiple comparisons
☐	☒	A full description of the statistical parameters including central tendency (e.g. means) or other basic estimates (e.g. regression coefficient) AND variation (e.g. standard deviation) or associated estimates of uncertainty (e.g. confidence intervals)
☐	☒	For null hypothesis testing, the test statistic (e.g. *F*, *t*, *r*) with confidence intervals, effect sizes, degrees of freedom and *P* value noted<br>*Give P values as exact values whenever suitable.*
☒	☐	For Bayesian analysis, information on the choice of priors and Markov chain Monte Carlo settings
☒	☐	For hierarchical and complex designs, identification of the appropriate level for tests and full reporting of outcomes
☒	☐	Estimates of effect sizes (e.g. Cohen's *d*, Pearson's *r*), indicating how they were calculated

Our web collection on statistics for biologists contains articles on many of the points above.

Software and code

Policy information about availability of computer code

Data collection	BD FACSDiva version 9 and Affymetrix Command Console (AGCC)v3.1.1.
Data analysis	FlowJo version 9.9 and 10, GraphPad Prism version 9, Morpheus Webtool and Transcriptome Analysis Console software v.4.0.3.

For manuscripts utilizing custom algorithms or software that are central to the research but not yet described in published literature, software must be made available to editors and reviewers. We strongly encourage code deposition in a community repository (e.g. GitHub). See the Nature Portfolio guidelines for submitting code & software for further information.

Data

Policy information about availability of data

All manuscripts must include a data availability statement. This statement should provide the following information, where applicable:

- Accession codes, unique identifiers, or web links for publicly available datasets
- A description of any restrictions on data availability
- For clinical datasets or third party data, please ensure that the statement adheres to our policy

The authors declare that all data supporting the findings of this study are available within the article and its supplementary information file.

Human research participants

Policy information about studies involving human research participants and Sex and Gender in Research.

Reporting on sex and gender	N/A
Population characteristics	N/A
Recruitment	N/A
Ethics oversight	N/A

Note that full information on the approval of the study protocol must also be provided in the manuscript.

Field-specific reporting

Please select the one below that is the best fit for your research. If you are not sure, read the appropriate sections before making your selection.

- [x] Life sciences
- [ ] Behavioural & social sciences
- [ ] Ecological, evolutionary & environmental sciences

For a reference copy of the document with all sections, see nature.com/documents/nr-reporting-summary-flat.pdf

Life sciences study design

All studies must disclose on these points even when the disclosure is negative.

Sample size	No statistical methods were used to pre-determine sample size, but our sample sizes (3-12 mice/group) are similar to those reported in our previous publications (https://doi.org/10.1084/jem.20151855 and https://doi.org/10.1016%2Fj.celrep.2019.10.126).
Data exclusions	No data were excluded from the analysis.
Replication	Data are representative of 2 or more experiments and all attempts at replication of the provided data were successful.
Randomization	Mice were allocated randomly into the indicated experimental groups.
Blinding	Experiments were not performed in a blinded manner.

Reporting for specific materials, systems and methods

We require information from authors about some types of materials, experimental systems and methods used in many studies. Here, indicate whether each material, system or method listed is relevant to your study. If you are not sure if a list item applies to your research, read the appropriate section before selecting a response.

Materials & experimental systems

n/a	Involved in the study	
☐	☒	Antibodies
☐	☒	Eukaryotic cell lines
☒	☐	Palaeontology and archaeology
☐	☒	Animals and other organisms
☒	☐	Clinical data
☒	☐	Dual use research of concern

Methods

n/a	Involved in the study	
☒	☐	ChIP-seq
☐	☒	Flow cytometry
☒	☐	MRI-based neuroimaging

Antibodies

Antibodies used	The following antibodies along with appropriate isotype controls were used in this study: CD45.2 PE/Cyanin-7, Pacific Blue or APC (1:400; Clone 104; BioLegend; Cat# 109830, 109820 or 109814), CD8a Brilliant Violet 711, Pacific Blue, APC, or BUV395 (1:400; Clone 53-6.7; BioLegend; Cat# 100759, 100725, 100711, or BD Biosciences Cat# 563786), CD44 Pacific Blue (1:400; Clone IM7; BioLegend; Cat# 103020), Thy1.1 PerCP/Cy5.5, Brilliant Violet 711, or Pacific Blue (1:1000; Clone OX7; BioLegend; Cat# 109004, 202539 or 202522), Thy1.2 Brilliant Violet 605 or Pacific Blue (1:1000; clone 53-2.1; BioLegend; Cat# 140318 or 140306), KLRG1 Violet Fluor 450 (1:200; Clone 2F1; Tonbo; Cat# 75-5893-U100), CD8b PerCP/Cy5.5 (Clone YST156.7.7; BioLegend; Cat# 126609), CD69 FITC or Pacific Blue (1:100; Clone H1.2FE, BioLegend; Cat# 104506 or or 104523), CD103 PE (1:200; Clone 2-E7; BioLegend; Cat# 121406), IFNg APC (1:200; Clone XMG1.2; BioLegend; Cat# 505810), PD-1 PE (1:100; Clone 29F1.1a12; BioLegend; Cat# 135206), ICOS FITC or PE (1:200;

	Clone 7E.17G9, eBioscience; Cat# 11-9942-82 or 12-9942-82), CXCR6 APC (1:300; Clone SA051D1, BioLegend; Cat# 151106), Blimp1 PE (1:200; Clone 5-E7, BioLegend; Cat# 150006), TCF-1 PE (1:200; Clone S33-966; BD Bioscience; Cat# 564217), Ki-67 PE/Cyanin7 (1:200; Clone 16A8, BioLegend; Cat# 652425), CD62L PE or APC (1:400; Clone MEL-14; BioLegend; Cat# 104408 or 104412), CD25 PE (1:200; Clone PC61; BioLegend; Cat# 102007) and Viability-Ghost Dye Red 780 (1:1000; Tonbo; Cat #13-0865-T100).
Validation	All antibodies used in this study were validated by the manufacturers for flow cytometry on mice. Relevant isotype controls were used as an additional validation for each antibody.

Eukaryotic cell lines

Policy information about cell lines and Sex and Gender in Research

Cell line source(s)	Primate renal epithelial cells (BSC-40) were purchased from ATCC and used for Vaccinia virus propagation and plaque assay.
Authentication	BSC-40 cells were not authenticated after purchase from ATCC
Mycoplasma contamination	Cells were negative for Mycoplasma contamination
Commonly misidentified lines (See ICLAC register)	No commonly misidentified lines were used in this study.

Animals and other research organisms

Policy information about studies involving animals; ARRIVE guidelines recommended for reporting animal research, and Sex and Gender in Research

Laboratory animals	All mice, including IFNg-YFP, used for this study were from strain C57BL/6 genetic background. Transgenic P14 and OT-I CD8 T cells were isolated from 12-14 weeks old female mice and adoptively transferred into age-matched femalemice.
Wild animals	The study did not involve wild animals.
Reporting on sex	Experiments that involved adoptive transfer of T cells were performed in females; all other experimental groups involved a mixture of males and females assigned randomly.
Field-collected samples	No field-collected samples were used in this study.
Ethics oversight	All animal procedures were approved by and performed in accordance with the Institutional Animal Care and Use Committee at OHSU.

Note that full information on the approval of the study protocol must also be provided in the manuscript.

Flow Cytometry

Plots

Confirm that:

- [x] The axis labels state the marker and fluorochrome used (e.g. CD4-FITC).
- [x] The axis scales are clearly visible. Include numbers along axes only for bottom left plot of group (a 'group' is an analysis of identical markers).
- [x] All plots are contour plots with outliers or pseudocolor plots.
- [x] A numerical value for number of cells or percentage (with statistics) is provided.

Methodology

Sample preparation	Cell staining and flow cytometry<br>Spleens of infected mice were harvested and single cell suspensions were generated by gently forcing the spleen through a mesh screen. Red blood cells were lysed by resuspending cell pellets in 150 mM NH4Cl, 10 mM KHCO3, and 0.1 mM Na-EDTA and staining for surface antigens was performed in PBS/1% FBS for 15 minutes at 4°C. Data was acquired using either a BD LSRII, BD Fortessa, or a BD Symphony Flow Cytometer using BD FACSDiva version 9 in the OHSU Flow Cytometry Core Facility. Flow cytometry data was analyzed using FlowJo software, version 9.9 or 10.<br><br>Ex vivo peptide stimulation and intracellular stain<br>Spleens of VacV-GP33 or VacV-SIINFEKL infected mice were harvested on the indicated day post-infection and single cell suspensions were generated as described in ‘Cell staining and flow cytometry’. For intracellular cytokine stain, splenocytes were seeded in a 96-well plate (2-3 million cells/well) and incubated with GP33-41 or SIINFEKL APL variants (Biosynthesis) in the presence of 1X Brefeldin A (BioLegend) for 5 hours at 37°C. Cells were then washed once and stained for surface antigens as described above followed by incubation with Cytofix/Cytoperm solution (BD Biosciences) for 30 minutes at 4° C. Cells were then washed with Perm/Wash Buffer (BD Biosciences), then incubated with IFNg antibody diluted in Perm/Wash Buffer for 30 minutes at 4°C, washed twice in Perm/Wash Buffer and resuspended in PBS/1%FBS for analysis by flow cytometry as described above. Intracellular staining for Blimp1 or TCF-1 was performed by incubating the cells in the Transcription Factor

	Fix/Perm Buffer (Tonbo Biosciences) for 45 minutes at 4°C followed by washing twice in Perm/Wash Buffer (BD Biosciences). Cells were incubated with the antibodies against Blimp1 or TCF-1 in Perm/Wash Buffer for 1 hour at 4°C followed by washing two more times with Perm/Wash Buffer. Cells were then resuspended in PBS/1% FCS for analysis by flow cytometry.
Instrument	Data was acquired using either a BD LSRII, BD Fortessa, or a BD Symphony Flow Cytometer
Software	BD FACSDiva was used for data acquisition and FlowJo version 9.9 and 10 were used for flow cytometric data analysis.
Cell population abundance	Sorted samples had purity > 95% as confirmed by re-sampling after sorting.
Gating strategy	SSC-A/FSC-A was used to gate on cells. Dead cells were excluded with Live/Dead Fixable Ghost dye. Doublets were excluded through FSC-W/FSC-A. Within the skin, hematopoietic cells were identified by gating on CD45 positive cells . Transferred P14 CD8 T cells were identified using the congenic marker Thy1.1 and gated as Thy1.1/CD8a double positive. A representative general gating strategy is depicted in Supplementary Figure 2a.

☒ Tick this box to confirm that a figure exemplifying the gating strategy is provided in the Supplementary Information.
